# 

**Published:** 1846-02

**Authors:** 


*Our Journal. Its proposed enlargement.*—It is our purpose, in accordance with the expressed desire of a large number of our subscribers, to commence our next volume in a new form, and of a size more adequate to the wants of our readers. The first number of our *third* volume will be issued as early in April as practicable, and will contain 96 pages. It will thereafter be issued *every other month* at $2.00 per annum in advance, thus giving to our patrons 576 pages in the volume, being *three times* the present amount of matter, for but *double* the present price of subscription. Our prospectus will be issued in a few days and will be sent to each of our subscribers, who are respectfully requested to aid us with their efforts and influence in extending our circulation, and thus enable us to defiay our greatly increased expenses. Our new volume will be printed with new type, upon good paper, and will be much improved in typographical execution, and we hope, in the interest and importance of its contents.—[Ed.

